# Data for increase of *Lymantria dispar* male survival after topical application of single-stranded RING domain fragment of IAP-3 gene of its nuclear polyhedrosis virus

**DOI:** 10.1016/j.dib.2016.03.007

**Published:** 2016-03-09

**Authors:** Volodymyr V. Oberemok, Kateryna V. Laikova, Aleksei S. Zaitsev, Vladimir A. Gushchin, Oleksii A. Skorokhod

**Affiliations:** a Vernadsky Crimean Federal University, Taurida Academy, Department of Biochemistry, Academician Vernadsky Ave., 4, 295007 Simferopol, Republic of Crimea; b Vernadsky Crimean Federal University, Medical Academy, Department of Biochemistry, Lenin Ave., 5/7, 295006 Simferopol, Republic of Crimea; c Lomonosov Moscow State University, Department of Virology, Moscow 119991, Russia; d University of Torino, Department of Oncology, via Santena 5 bis, Torino 10126, Italy

**Keywords:** Pest management, Gypsy moth Lymantria dispar, DNA insecticides, Lymantria dispar multicapsid nuclear polyhedrosis virus, Viral IAP genes, RING (really interesting new gene), Insecticide resistance

## Abstract

This data article is related to the research article entitled “The RING for gypsy moth control: topical application of fragment of its nuclear polyhedrosis virus anti-apoptosis gene as insecticide” [1]. This article reports on significantly higher survival of gypsy moth *Lymantria dispar* male individuals in response to topical application of single-stranded DNA, based on RING (really interesting new gene) domain fragment of LdMNPV (*L. dispar* multicapsid nuclear polyhedrosis virus) IAP-3 (inhibitor of apoptosis) gene and acted as DNA insecticide.

**Table:** 

Subject area	Biology
More specific subject area	Pesticide biochemistry and physiology, plant protection, creation of insecticides
Type of data	Histogram
How data was acquired	Count of survived male individuals
Data format	Processed data
Experimental factors	*Lymantria dispar* larvae were reared on *Quercus robur* leaves in the laboratory until imago stage. The count of survived male individuals was performed for parameter assessment.
Experimental features	Distinction of male and female imago individuals is based on morphological difference.
Data source location	V.I. Vernadsky Crimean Federal University, Simferopol, Republic of Crimea
Data accessibility	Data are provided with this article

## 1 **Value of the data**

• The data for the first time show that topical application of single-stranded DNA from RING domain of LdMNPV IAP-3 gene leads to the significantly higher survival of *Lymantria dispar* male imago individuals, preferentially targeting the female imago individuals.
• The data, in our opinion, describe the consequence for previously observed decreased accumulation of biomass of caterpillars, miss-regulated expression of apoptosis and anti-apoptosis genes, calcium and magnesium imbalance and increased alkaline phosphatase activity in gypsy moth cells after treatment with RING domain fragment which targeted female individuals stronger than males [1].
• Data on selective survival of *Lymantria dispar* male individuals in response to RING DNA insecticide may be of interest for plant protection approaches.
• The data support the concept that DNA insecticides could have the number of advantages in creation of insecticides based on nucleic acids in comparison with RNA interference approach [1], [2], [3] and could resolve or improve insecticide resistance problem [1], [4].

## 2 Data

**Fig. 1:**
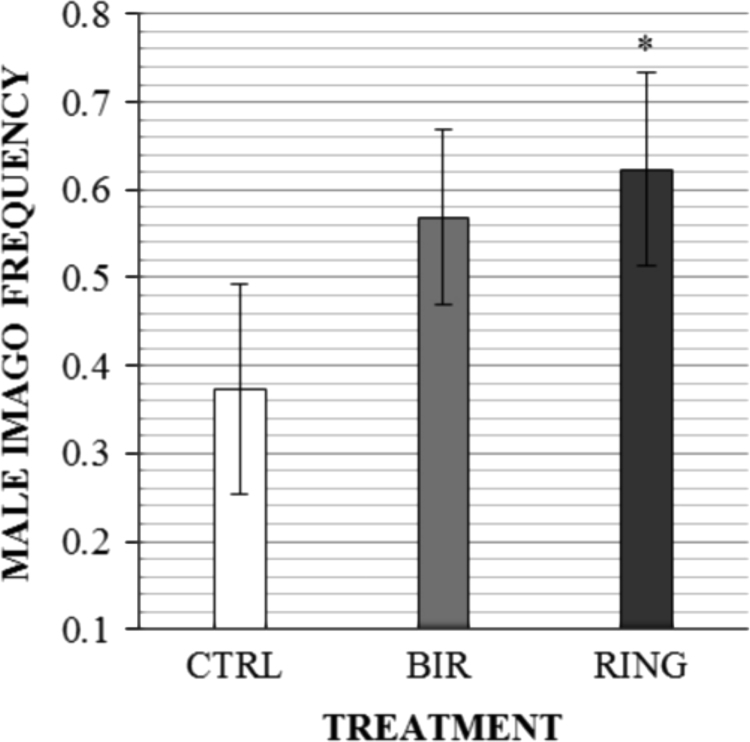
The frequency of male gypsy moths survived after DNA insecticide treatment in control (CTRL), BIR (baculoviral IAP (inhibitor of apoptosis) repeat) and RING (really interesting new gene) groups. Mean and standard errors are presented. Significance of difference versus CTRL is indicated by ^*^ for *p*<0.05 (chi-squared test *χ*^2^ value is 4.09).

Data for *Lymantria dispar* male survival after topical application of different single-stranded DNA (ssDNA) fragments are presented in Fig. 1 as survived male imago frequency.

## 3 Experimental design, materials and methods

### 3.1 Treatment technique

In average, 20–25 2nd instar caterpillars from each of three Crimean locations were used per each control and experimental groups for the treatment with ssDNA (for details see [1] and the section “Sequences of the applied DNA fragments”). Each experiment was performed in 4 replicates (thus 80–100 caterpillars were included for each treatment group). A water solution with ssDNA (10 pmol/μl, either BIR or RING) was applied topically on caterpillars via fine spraying (2–3 pmol of ssDNA per caterpillar) [1].

### 3.2 Insect rearing

Control and treated with ssDNA gypsy moth caterpillars were grown in Petri dishes on oak leaves (*Quercus robur*) at temperature 25 °C until pupation. On emergence of imago from pupae, the numbers of adult male and female moths were counted [1].

### 3.3 Sequences of the applied DNA fragments

We designed DNA fragments as described in [1], [2], [5], [6]. DNA fragments were synthesized by Metabion International AG (Germany). The sequences of the applied single-stranded DNA fragments were the following: (1) 5′-GCC GGC GGA ACT GGC CCA-3′ (134843–134860; sense strand; BIR domain; control group) and (2) 5′-CGA CGT GGT GGC ACG GCG-3′ (135159135142; antisense strand; RING domain; experimental group).

### 3.4 Statistical analysis

Non-parametric Pearson׳s chi-squared test (*χ*^2^) and Mann–Whitney test to evaluate the significance of difference between the groups’ means (Sofa Statistics 1.3.3 software) were applied.
